# Effect of CeO_2_ Nanoparticles on Interface of Cu/Al_2_O_3_ Ceramic Clad Composites

**DOI:** 10.3390/ma13051240

**Published:** 2020-03-09

**Authors:** YaBo Fu, HaoNan Chen, ZhiQiang Cao, YanQiu Huo

**Affiliations:** 1 Zhejiang Provincial Key Laboratory for Cutting Tools, School of Pharmaceutical and Materials Engineering, Taizhou University, Taizhou 318000, China; Chenhaonan2333@163.com (H.C.); yqhuo@tzc.edu.cn (Y.H.); 2 School of Materials Science and Engineering, Dalian University of Technology, Dalian 116024, China; caozq@dlut.edu.cn

**Keywords:** CeO_2_ nanoparticles, bonded strength, Al_2_O_3_ ceramic, CeO_2_–Cu_2_O–Cu

## Abstract

Cu/Al_2_O_3_ ceramic clad composites are widely used in electronic packaging and electrical contacts. However, the conductivity and strength of the interfacial layer are not fit for the demands. So CeO_2_ nanoparticles 24.3 nm in size, coated on Al_2_O_3_ ceramic, promote a novel CeO_2_–Cu_2_O–Cu system to improve the interfacial bonded strength. Results show that the atom content of O is increased to approximately 30% with the addition of CeO_2_ nanoparticles compared with the atom content without CeO_2_ in the interfacial layer of Cu/Al_2_O_3_ ceramic clad composites. CeO_2_ nanoparticles coated on the surface of Al_2_O_3_ ceramics can easily diffuse into the metallic Cu layer. CeO_2_ nanoparticles can accelerate to form the eutectic liquid of Cu_2_O–Cu as they have strong functions of storing and releasing O at an Ar pressure of 0.12 MPa. The addition of CeO_2_ nanoparticles is beneficial for promoting the bonded strength of the Cu/Al_2_O_3_ ceramic clad composites. The bonded strength of the interface coated with nanoparticles of CeO_2_ is increased to 20.8% compared with that without CeO_2_; moreover, the electric conductivity on the side of metallic Cu is 95% IACS. The study is of great significance for improving properties of Cu/Al_2_O_3_ ceramic clad composites.

## 1. Introduction

Cu/Al_2_O_3_ ceramic clad composites have anti-wear, anti-corrosion, and anti-high temperature characteristics of ceramics and maintain the high conductivity and machinability of copper. They have been widely used in rail transit, electronic packaging, and electrical contacts [1,2]. Although Cu/Al_2_O_3_ ceramic clad composites have the advantages of a ceramic and a copper, ceramic is brittle and difficult to process. Assembly and connection structures of ceramic and metal are often used. To obtain a stable and reliable Cu/Al_2_O_3_ ceramic clad structure, the wettability between metals and ceramics and the formation of brittle compounds at the interface must be addressed. These problem are of great research significance [3,4].

Many researchers have carried out the research. At present, brazing and diffusion bonding are the main methods to achieve connections between ceramics and metals. Breslin et al. [5] have suggested that the key problem was the interfacial wettability between the ceramics and metals, so a method of co-continuous ceramic composites was proposed [6,7]. The surface of ceramics coated a layer of Mo–Mn could improve wettability [8]. Active metals, such as Ag, Ti, Zr and V, have been added to study effects. However, general oxygen content was lower than 1 Pa to avoid oxidizing [9,10,11]. Burgess et al. [12,13] have used a Cu-Cu_2_O eutectic liquid system to bond Cu layers with ceramics for the first time. However, Fan Jinglian et al. [14] have discovered that wettability between Cu and Al_2_O_3_ was still not significantly improved when the temperature was increased from 1200 to 1400 °C.

Results [15,16] have shown that the contact angle between the molten Cu and Al_2_O_3_ ceramic was 158°–170° under oxygen-free conditions at 1100–1300 °C, so they were non-wetting each other. Diemer et al. [17] have found that by controlling the oxygen partial pressure (p_O2_) and oxygen content in the copper simultaneously, contact angle could be varied between 125°and 22°. Evaluation of the Gibbs adsorption equation for the liquid/solid interface at 1300 °C suggests that adsorption of a Cu–O complex at that interface plays a key role in promoting wetting. Formation of CuAlO_2_ and dissolution of Al_2_O_3_ in the melt also influence the contact angle, especially in the range of p_O2_ > 1 Pa. When the content of O was higher than 2 at.%, Cu began wetting the Al_2_O_3_. Huang [18] and Chatterjee [19] have found that the addition of oxides to metal solders could improve the wettability between Al_2_O_3_ ceramics and Cu layers. However, there are few reports on improving bonded strength between Cu and Al_2_O_3_ ceramics by the addition of rare earth oxides and reducing the bonded temperature. Thus, new methods are needed to solve these problems.

In this study, CeO_2_ nanoparticles coat the interface between Al_2_O_3_ ceramics and Cu to form a new CeO_2_–Cu_2_O–Cu system to increase interfacial strength. Under a special gas pressure and temperature, the poor strengths of the ceramic/copper composites will be improved. The new phases and elements diffusing at low temperatures are studied. A new Cu/Al_2_O_3_ ceramic clad composite with the addition of CeO_2_ is fabricated.

## 2. Materials and Methods

**Figure 1:**
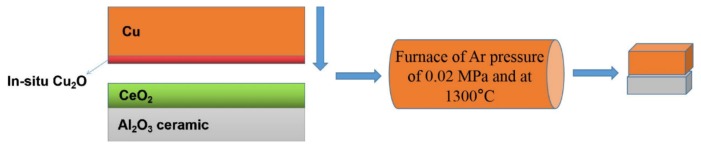
Sketch of experimental methods.

The specimens were prepared in a vacuum tube furnace (Boyun Tong company, Nanjing, China), which was vacuumed to 0.01 MPa and then filled Ar gas at a pressure of 0.12 MPa. The Cu cubes (Zhejiang wanteng metal materials firm, Ningbo, China) were 99.90 wt.% Cu. The ceramic cubes (Shenzhen beilong electronic material factory, Shenzhen, China) were 99.9 wt.% Al_2_O_3_. The fabrication procedures were as follows: nanoparticles of CeO_2_ coated the surface of Al_2_O_3_ ceramics → in situ Cu_2_O formed in the Cu interface in the 40 °C air → the melting of Cu and Al_2_O_3_ ceramic clad composites at 1300 °C for 5 min in a furnace at an Ar pressure of 0.12 MPa → a cube with the dimensions 40 × 40 × 30 mm was formed, as shown in Figure 1.

After preparation, the samples were etched in a solution containing 3 g of FeCl_3_, 2 mL of HCl, and 96 mL of C_2_H_5_OH. Their metallurgical structures and microstructures were examined by scanning electron microscopy (SEM S-4800, Hitachi, Tokyo, Japan), and backscattered electron imaging (BSE, Hitachi, Tokyo, Japan) under a control voltage of 20 kV. The electric conductivity was measured at 60 kHz using a digital portable eddy current tester (FD-102, Xiamen xinrui instrument Ltd., Xiamen, China). The bonded strength was measured with a nanomechanical test for Nano Test 600 (Micro Mmaterials Ltd., Wrexham, UK). XRD patterns were obtained using a Bruker D8 Advance (Bruker Ltd., Karlsruhe, Germany) with Cu Kα radiation.

## 3. Results and Discussion

### 3.1. Structure and Hardness of Cu/Al_2_O_3_ Clad Composites

**Figure 2:**
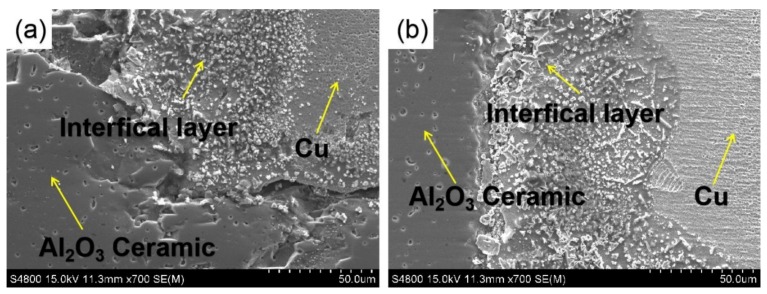
SEM images of Cu/Al_2_O_3_ composites at bonded temperature 1300 °C: (**a**) without CeO_2_ and (**b**) with CeO_2_.

**Figure 3:**
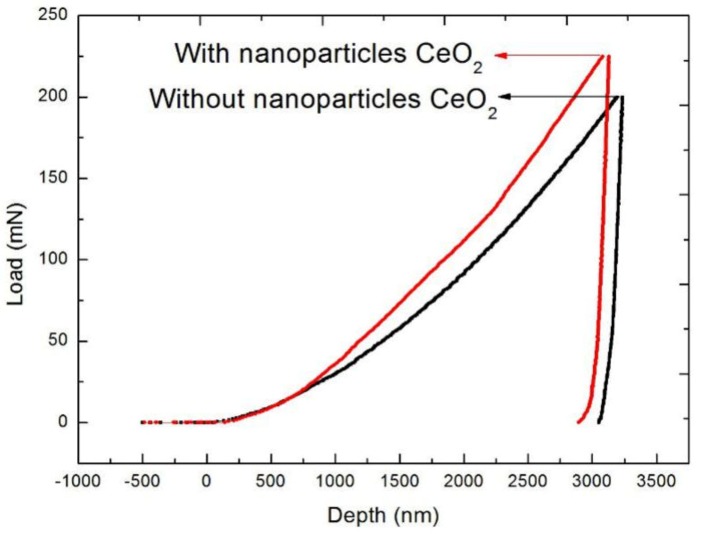
Bonded strength of interfacial layer.

Figure 2 shows the SEM images of Cu/Al_2_O_3_ composites at bonded temperature of 1300 °C. When nanosized CeO_2_ is not added, the metal Cu and Al_2_O_3_ ceramic could not form a new eutectic solution. The wettability of the two ceramics was poor, so there are many cracks in the bonded interface at 1300 °C, as shown in Figure 2a. The measurements are taken at room temperature. However, a closely bonded interface is formed between the Cu and Al_2_O_3_ by addition of CeO_2_ nanoparticles, as shown in Figure 2b. There are no cracks in the bonded interface at 1300 °C. The conductivity of the Al_2_O_3_ ceramic is 0% IACS (international annealed copper standard), whereas the conductivity of the side of metallic Cu is 95% IACS, so the conductivity of Cu in the clad composites is reserved. Figure 3 shows the bonded strengths of the interfacial layer of Cu/Al_2_O_3_ clad composites with CeO_2_ and without CeO_2_ at the bonded temperature 1300 °C. The bonded strength of Cu/Al_2_O_3_ interfacial layer with nanoparticles of CeO_2_ is 990.3 MPa; however, that without CeO_2_ is 820.1 MPa. The bonded strength of the interfacial layer coated with nanoparticles of CeO_2_ increases 20.8% compared with that without CeO_2_. Therefore, nanoparticles of CeO_2_ can improve the bonded strength.

### 3.2. EDS of Interface

**Figure 4:**
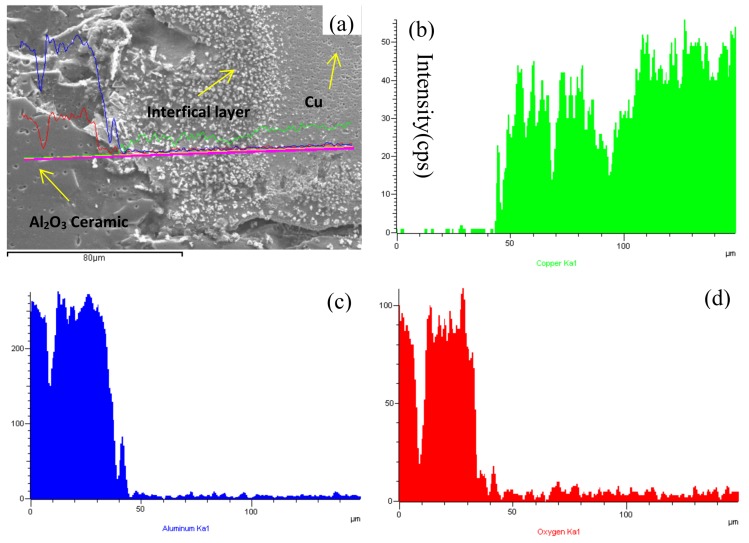
Energy-dispersive X-ray spectroscopy (EDS) of Cu/Al_2_O_3_ ceramic interface without CeO_2_: (**a**) interface, (**b**) Cu, (**c**) Al, (**d**) O.

The element distributions of the interfaces of the composite materials are presented in this section. Figure 4 shows the energy-dispersive X-ray spectroscopy (EDS) of the Cu/Al_2_O_3_ interface without CeO_2_. In the range of 0–40 μm, the contents of Al and O are high, whereas the content of Cu is the lowest, which indicate that 40 μm is the dividing line of the interface of the clad composites. However, in the range of 40–150 μm, the content of Cu is the highest, and a small amount of Al and O can diffuse to the copper layer.

**Figure 5:**
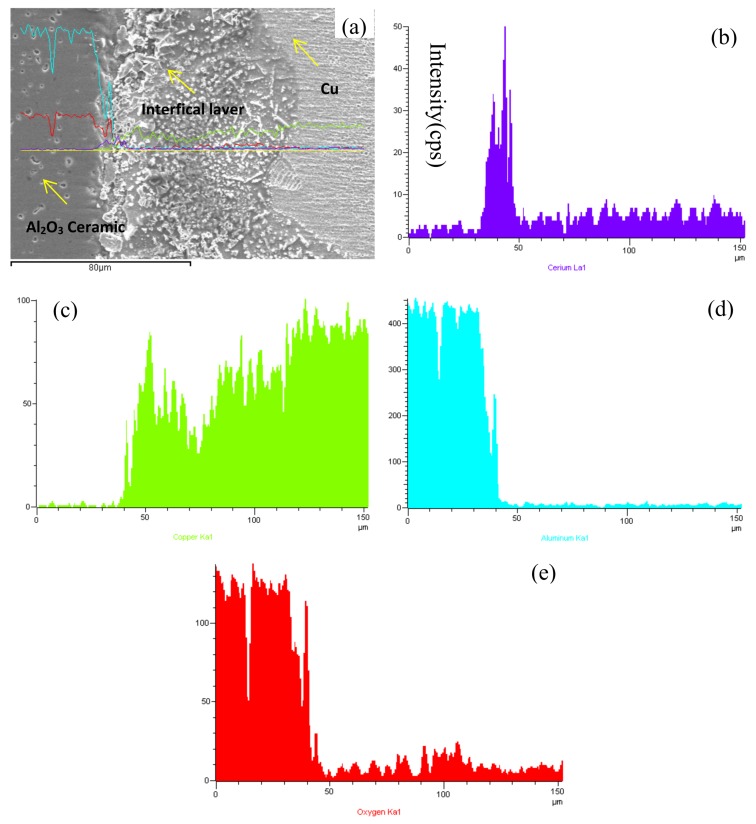
EDS of interface coated with CeO_2_: (**a**) interface, (**b**) Ce, (**c**) Cu, (**d**) Al, (**e**) O.

On the bonding surface coated with nanoparticles of CeO_2_, the contents of these elements are different from that without CeO_2_, as shown in Figure 5. In the range of 0–40 μm, the contents of Al and O are higher than Cu, whereas the content of Cu is the lowest in all. This indicates that 40 μm is the dividing line of the interface for the clad composites. The content of O is significantly increased to 30% with addition of CeO_2_ compared with that without CeO_2._, which indicate that CeO_2_ could raise the content of O in the interface. The atom content of O tested by EDS is 15.4% when CeO_2_ nanoparticles are not coated in the surface of Al_2_O_3_ ceramic; however, the atom content of O is increased to 20.4% when CeO_2_ nanoparticles are coated. Again, the results prove that the atom content of O is 30% higher compared with that without CeO_2._

Moreover, the content of Ce arises from the range of 0–150 μm due to the addition of CeO_2_. Figure 5b shows that the CeO_2_ coated on the surface of the alumina ceramics can easily diffuse into the metallic Cu layer, but not to the Al_2_O_3_ ceramics. O and Cu easily form a eutectic liquid of Cu_2_O–Cu under certain conditions [12,13], so CeO_2_ can accelerate to form the eutectic liquid of Cu_2_O–Cu by storing or releasing O at Ar pressure of 0.12 MPa. However, in the range of 40–150 μm, the content of Cu is the highest, and the contents of O and Al are reduced. The eutectic liquid of Cu_2_O–Cu is the key factor in improving the wettabilities of the Cu and Al_2_O_3_ layers.

### 3.3. Mechanisms Discussion

**Figure 6:**
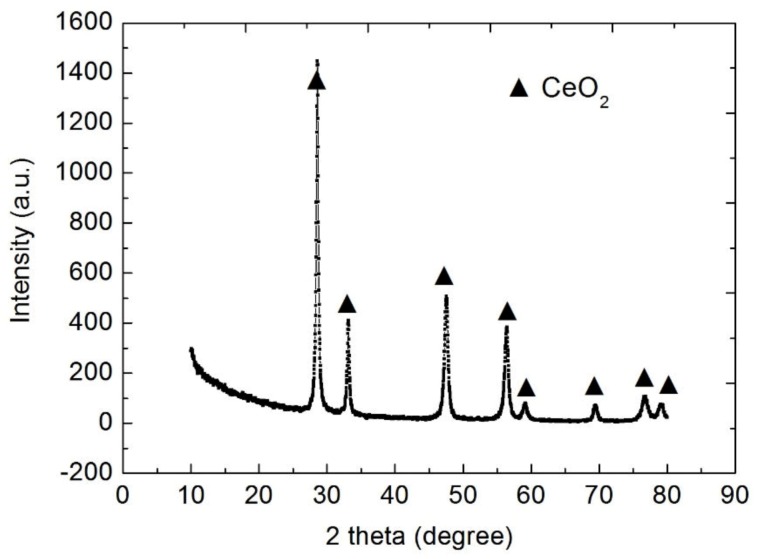
XRD patterns of CeO_2_.

**Figure 7:**
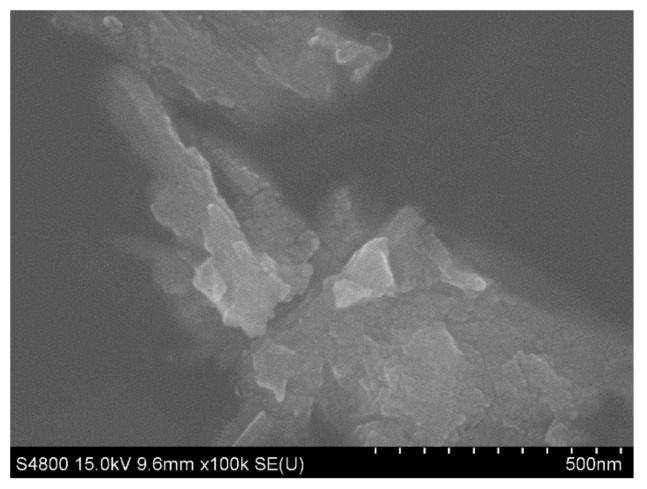
SEM image of CeO_2_ nanoparticles.

**Figure 8:**
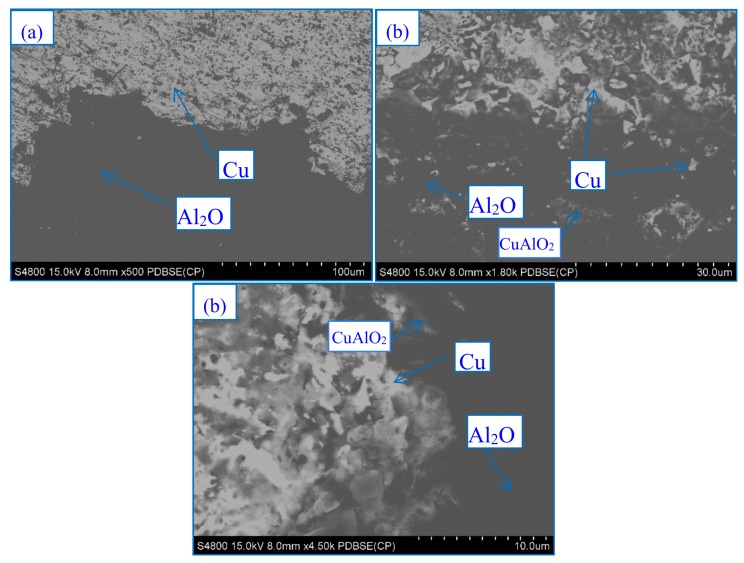
Backscattered electron imaging (BSE) images at samples’ bonded temperatures: (**a**) without CeO_2_ at bonded temperature 1300 °C, (**b**) without CeO_2_ at bonded temperature 1500 °C, and (**c**) with CeO_2_ at bonded temperature 1300 °C.

Figure 6 shows the XRD patterns of the nanoparticles of CeO_2_, the size of which are 24.3 nm for 28.5°according to the Peak Search Report of XRD. Thus, rare earth oxide coated on the surface of Al_2_O_3_ ceramics is CeO_2_. Figure 7 shows the SEM image of CeO_2_. Due to the presence of nanoparticles of CeO_2_, elements are active and easily diffused into the layers, which is beneficial for reducing the bonding temperature. Figure 8 shows the BSE images of the interface at bonded temperatures of 1300 and 1500 °C. At 1500 °C, Al and Cu easily diffuse into each other to form the compound of CuAlO_2_. Which is beneficial to the improvement of wettability [8]. At 1300 °C, the diffusion rates of the elements are decreased and oxidation cannot occur in time; however, the interface coated with CeO_2_ shows that Al and Cu can diffuse into each other quickly in 5 min. Consequently, slags and cracks cannot form at low temperatures. The results show that the addition of nanoparticles CeO_2_ is beneficial for reducing the bonding temperature.

Al_2_O_3_ and Cu usually do not wet each other. The contact angle between the molten Cu and Al_2_O_3_ ceramic is 158°–170° under oxygen-free conditions at 1100–1300 °C [15,16]. Thus, the Cu and Al_2_O_3_ are completely non-wetting when the Cu is molten. To achieve excellent properties of Cu/Al_2_O_3_ clad composite, it is necessary to improve the wettability between them. A small amount of O could reduce the wetting angle between Cu and Al_2_O_3_ [17]. When the content of O is higher than 2 at.%, Cu begins wetting the Al_2_O_3_. Moreover, Cu and Cu_2_O could form a eutectic liquid [20]. However, CuO is easily formed at this temperature, so its formation must be strictly prevented. Therefore, reducing the content of O to prevent the formation of CuO and promoting the formation of Cu–Cu_2_O are the key factors. In this study, CeO_2_ can react with copper to form Cu_2_O instead of CuO, promoting the formation of the Cu–Cu_2_O eutectic solution. Yang, Y.M. [21] declared that in CeO_2_ crystal structure Ce^+4^ was easily converted to Ce^+3^, or vice versa, which makes the nanoparticles of CeO_2_ have strong functions of storing and releasing oxygen, as shown in chemical Equation (1). Chemical Equation (2) shows that Cu_2_O can be formed under the condition of trace oxygen content. The chemical equations are as follows:

CeO_2_ ↔ CeO_2(1−X)_ + xO_2_ (0 ≤ x ≤ 0.25) (1)

Cu+O_2_ → Cu_2_O (2)

The reduction of CeO_2_ to Ce_2_O_3_ has been reported in the high temperature sintering of fine CeO_2_ particles by [22,23]. The authors in reference [24] have also proved the conclusion by High Resolution Transmission Electron Microscopy (HRTEM) imaging and Fast Fourier Transformation (FFT) diffraction. Thus, nanoparticles of CeO_2_ can improve the bonded strength, as CeO_2_ nanoparticles have strong functions of oxygen storage and release and, thus, the addition of CeO_2_ nanoparticles play an important role for a novel system of CeO_2_–Cu_2_O–Cu at an Ar pressure of 0.12 MPa.

The authors in [17] reported that, due to the mutual diffusion and redistribution of chemicals in the melting process, these oxides react with Al_2_O_3_ under certain conditions to form CuAlO_2_, which is beneficial to the improvement of wettability [8]. The corresponding Gibbs free energies (ΔG^0^) [25] are as follows:

2Cu_2_O + O_2_ → 4CuO     ΔG^0^ = −62354 + 44.89 T (3)

2Cu_2_O + O_2_ → 4CuO     ΔG^0^ = −62354 + 44.89 T (4)

Equations (3) and (4) show that the Gibbs free energies of the above reactions are negative at 1300 °C, so CeO_2(1−X)_ can absorb a small amount of O_2_ to prevent CuO formation, and CuAlO_2_ can also be formed spontaneously.

**Figure 9:**
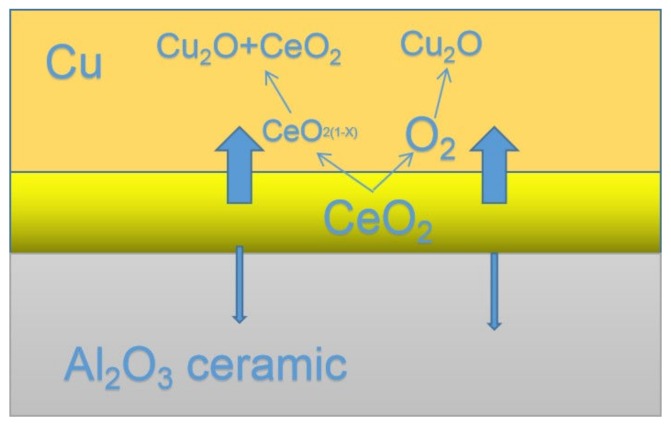
Mechanism of Cu-Cu_2_O-CeO_2_ eutectic liquid formation at bonded temperature 1300 °C.

**Figure 10:**
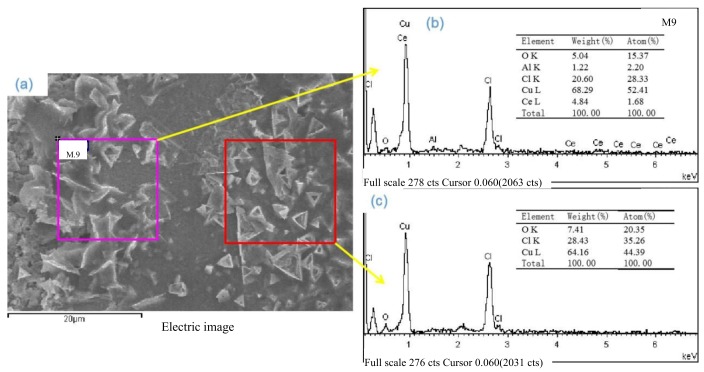
SEM images and EDS of interface (Cl is the etched residuum): (**a**) SEM, (**b**) ceramic side, (**c**) Cu side.

The reaction temperature of 1300 °C is 65 °C higher than the melting point of Cu_2_O (1235 °C). Thus, Cu_2_O reacts rapidly with Al_2_O_3_ to form compounds of CuAlO_2_ at the interface in only 5 min. The formation of these low melting point copper oxides and interfacial compounds (CuAlO_2_) is beneficial for the liquid phase copper wetting of the alumina ceramic (melting point of CuO is 1200 °C). Under certain conditions, CeO_2_ is decomposed at 1000 °C and easily diffuses to the Cu layer, accelerating the formation of the CeO_2_–Cu_2_O–Cu eutectic, as shown in Figure 9. Figure 10 shows the SEM images and EDS of the interface. A strong structure, which is the key to strengthening the bonding interface, is formed due to the triangular type of the Cu_2_O, and Y type of CeO_2_ and CuAlO_2_.

## 4. Conclusions

(1) The atom content of O is increased to approximately 30% with addition of CeO_2_ nanoparticles 24.3 nm in size compared with the atom content without CeO_2_ nanoparticles in the interfacial layer of the Cu/Al_2_O_3_ ceramic clad composites, so the addition of CeO_2_ could raise the atom content of O;
(2) CeO_2_ nanoparticles coated on the surface of the Al_2_O_3_ ceramics can easily diffuse into the metallic Cu layer, but they do not in Al_2_O_3_ ceramics. CeO_2_ nanoparticles can accelerate to form the eutectic liquid of Cu_2_O–Cu, as they have strong functions of storing and releasing O at an Ar pressure of 0.12 MPa;
(3) The addition of CeO_2_ nanoparticles is beneficial for promoting the bonded strength of Cu/Al_2_O_3_ ceramic clad composites. The bonded strength of the interface coated with nanoparticles of CeO_2_ is 20.8% higher than that without CeO_2_; however, the electric conductivity of metallic Cu is 95% IACS.
